# Recent Impact of Microfluidics on Skin Models for Perspiration Simulation

**DOI:** 10.3390/membranes11020150

**Published:** 2021-02-21

**Authors:** Genís Rabost-Garcia, Josep Farré-Lladós, Jasmina Casals-Terré

**Affiliations:** 1 Department of Mechanical Engineering, MicroTech Lab, Universitat Politècnica de Catalunya (UPC), C/Colom 7-11, 08222 Terrassa, Spain; josep.farre.llados@upc.edu (J.F.-L.); jasmina.casals@upc.edu (J.C.-T.); 2 Onalabs Inno-hub S.L., C/de la Llibertat 11, 08012 Barcelona, Spain

**Keywords:** skin models, skin phantom, artificial skin, perspiration, sweat, wearables, in vitro testing

## Abstract

Skin models offer an in vitro alternative to human trials without their high costs, variability, and ethical issues. Perspiration models, in particular, have gained relevance lately due to the rise of sweat analysis and wearable technology. The predominant approach to replicate the key features of perspiration (sweat gland dimensions, sweat rates, and skin surface characteristics) is to use laser-machined membranes. Although they work effectively, they present some limitations at the time of replicating sweat gland dimensions. Alternative strategies in terms of fabrication and materials have also showed similar challenges. Additional research is necessary to implement a standardized, simple, and accurate model representing sweating for wearable sensors testing.

## 1. Introduction

**Figure 1:**
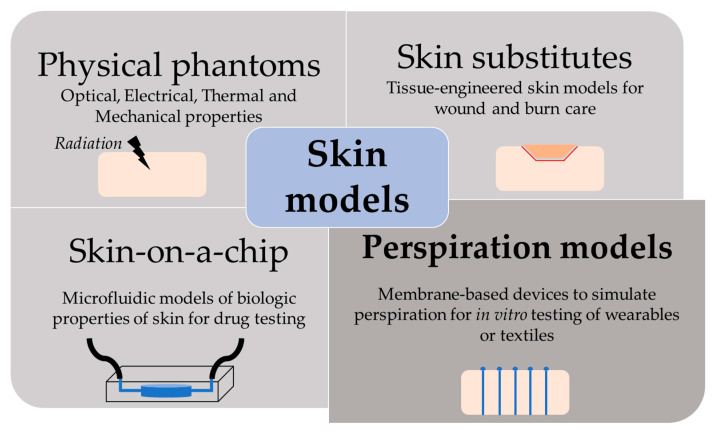
Scheme of the different purpose and applications of skin models.

The skin is our first interface with the surrounding environment, simultaneously acting as a protective barrier and a sensing platform. Skin studies are necessary to assess its real nature despite the limitations that experimentation on living tissue present. Researchers have been using non-invasive methods for the past decades, with the use of skin replica methods being a well-known example for the study of surface microtopography [1]. In recent years, there has been an increase in the implementation of in vitro models at multiple levels. This way, ethical issues and the high variability of human tests are avoided, while speeding up the testing process thanks to their longer storage stability and lower costs [2]. We have divided the skin models found in the literature into four main groups, depending on their purpose: physical phantoms, skin substitutes, skin-on-a-chip, and perspiration models (Figure 1). 

Physical models are based on biologically inactive materials that reproduce specific skin properties or behavior [2]. Here, we have englobed the replication of optical, electrical, thermal, and mechanical properties. The nature of phantoms is highly variable. Taking optical properties as an example, physical models can range from a purely optoelectronic setup (LED array) for optimization of photoplethysmographic signals [3] to liquid suspensions with added particles to mimic scattering and absorption characteristics of skin [4]. Polymeric materials offer a wide range of tunable properties, with the capability of adding fillers to tailor their characteristics even better [2]. Natural polymers, such as gelatin or agar, have been used for replicating tissue elasticity [5], the temperature distribution during laser irradiation [6], the bioimpedimetric response of skin [7], or the interaction with electromagnetic waves [8]. The use of synthetic polymers is also extended, highlighting elastomeric materials such as silicones due to their similar mechanical properties [9] or epoxy resins for their thermal [10] and optical [11] similitudes to skin. 

Tissue-engineered skin substitutes are mostly needed for full-thickness injuries, where all skin layers are affected, and skin grafting is required as they cannot epithelialize on their own [12]. Tissue donation is painful, with limited availability, and it may lead to severe health complications. Therefore, an off-the-shelf alternative capable of protecting and repairing the injured skin is of high interest. Typically, skin substitutes consist of a biocompatible and bioabsorbable scaffold, which can contain human cells and growing factors to promote skin regeneration [13].

Skin-on-a-chip models were developed to overcome the limitations of two-dimensional (2D) cell cultures and animal models in drug testing [14]. Conventional 2D cell cultures have well-established study conditions but fail to replicate the complex interaction between cells and the extracellular matrix. Animal models are both time- and resource-consuming, involve ethical issues, and may not reflect the nature of the human organism. A promising solution is merging novel three-dimensional culturing techniques, which would better represent the interactions of in vivo conditions, and microfluidic platforms for an accurate control of the physical and biochemical parameters of the system [15].

This review focuses on perspiration models. Similar to the previous models, there are multiple options to simulate sweating depending on the specific purpose of the model. There is extensive research on the thermal interaction between the human body, clothing, and the environment. In this field, sweating is considered as a key parameter, given that its main function of thermoregulating the body temperature [16]. The simplest models consist in a flat plate or a cylindric surface such a porous sintered metal heated up to 35 ºC and used as sweat generator (ISO 11092:2014) [17]. Such systems often use metals and have limited replication of mechanical properties and thermal inertia of skin [2]. They also lack the capability to be applied in actual clothing systems in use, which promoted the expansion of thermal manikins [18]. 

Thermal manikins have significantly evolved, from non-sweating and non-movable [19] to sweaty and movable [20], and have even been coupled with thermoregulation physiological models [21]. Sweating can be simulated by supplying water through tiny tubes to holes distributed at the manikin surface, as in the study performed by Li et al. [22], where 187 sweat glands are individually controlled through a complex system of microvalves. These manikins are highly costly due to the intricate heating and sweating supplies, limiting the sweating simulation to a manageable number of tubes and holes, reducing the accuracy of the model. Other manikins such as Walter [20] use a waterproof and breathable fabric to simulate skin that contains water in circulation, which emulates blood circulation. This allows for homogenous vapor perspiration over the entire manikin to be achieved, while reducing the complexity of the control system. 

However, in recent years, the study of sweat has raised interest as a rich biofluid containing useful information about subject health conditions [23]. These include disease biomarkers, such as the well-known case of cystic fibrosis diagnosis [24], metabolites of interest [25], or drugs [26]. While the possibility of a non-invasive alternative to blood is appealing, more research is needed to determine the potential correlations between sweat and blood [16]. 

With this aim, there is recent research on wearable devices combining sensors, microfluidics, and electronics [27,28,29]. Yet, these devices are currently still at the research phase and human validation is both time- and resource-consuming, and has a high degree of variability [30], which limits the comparison between studies. A standard, easy, and cost-effective in vitro evaluation of sweat wearable prototypes can enhance the development of these technologies by providing rapid and accurate feedback at the prototyping stage. 

This paper reviews the solutions proposed regarding perspiration models. First, an overview of the skin characteristics of interest, which include sweat gland dimensions and density, to replicate surface skin characteristics such as wettability, roughness, and texture. This paper also presents the technical challenges in the fabrication of these models and how they were addressed.

## 2. Skin and Perspiration Properties

This section reviews the parameters and properties of skin relevant to perspiration models in order to specify the requirements for this particular function. For perspiration simulation, bulk mechanical, optical, or electrical properties are not parameters of interest. On the other hand, sweat gland characteristics (dimensions and their density), surface skin properties (roughness, skin texture, and wettability), and sweat rates are key parameters to be replicated. 

**Figure 2:**
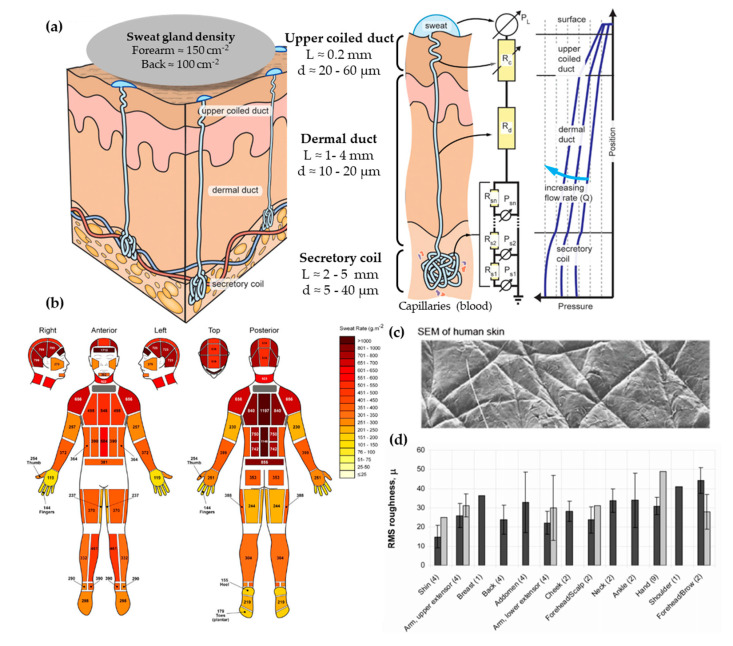
(**a**) Scheme showing human eccrine sweat gland structure and its microfluidic equivalent model. Adapted with permission from [32]. Copyright 2015, AIP. (**b**) Absolute regional median sweat rates of male athletes at a fixed exercise intensity (75% VO_2_) measured gravimetrically using absorbent pads. Reprinted with permission from [33]. Copyright 2010, Springer Nature. (**c**) SEM photography of human skin. Adapted with permission from [34]. Copyright 2013, Royal Society of Chemistry. (**d**) In vivo skin roughness, RMS values, obtained by a speckle device (black) and fringe projection systems (clear). Reprinted with permission from [35]. Copyright 2010, Intechopen.

From the three types of sweat glands that can be found in skin, there is more interest in the morphology and physiology of eccrine sweat glands. These can be found across the body’s surface and they are responsible for secreting eccrine sweat, which is hereinafter referred to as sweat. Apocrine and apoeccrine sweat glands are located on certain hairy regions of the body, such as the axilla or genital region [31], and they are not reviewed in this article. The eccrine gland is a coiled single tubular structure emerging from the deep dermis that can have a total length of 4 to 8 mm and can be divided into three distinct parts: the secretory coil, dermal duct, and upper coiled duct (Figure 2a) [32]. The secretory coil is the bundled coil where sweat is generated and osmotically pumped to the skin surface through the ductal regions, which have reabsorption mechanisms of sodium and chloride ions. 

The sweat generation mechanism has an important effect on each biomarker partitioning and correlation with blood levels, as detailed by Sonner et al. [32]. The process takes place in the secretory coil and it starts with signals from the brain that stimulate cholinergic nerve endings, the target of the perspiration stimulating agents, such as pilocarpine or carbachol [36]. This signal initiates a cascade of ions that develop a chemical and electric gradient, resulting in a higher osmolality in the lumen of the secretory coil than in the surrounding cells. This imbalance causes a water influx into the lumen to re-equilibrate concentrations, creating an osmotic pressure (Equation (1), Van’t Hoff’s law) that pumps the fluid into the ductal region. Schulz et al. [37] obtained experimental data of the osmotic pressure generated by sweat glands through inserting a micropipette with an incorporated manometer directly in a sweat duct. They reported a broad range of secretory pressures, from 3 kPa to a maximum of 70 kPa, with mean values around 40 kPa. These values are in agreement with the ones obtained using Van’t Hoff’s Law assuming ideal conditions [32].

P = σRTΔC(1)

where P is the pressure differential created, σ is the osmotic reflection coefficient, R is the gas constant, T is the temperature of the body, and ΔC is the difference in concentration between plasma and sweat usually represented in terms of osmolality.

Regarding the dimensions of the sweat gland, the secretory coil has an inner diameter between 5 and 40 µm with a length up to 5 mm due to its coiled structure. The straight dermal duct has an inner diameter of 10–20 µm with a slight enlargement at the upper coiled duct (20–60 µm). The total ductal length ranges from 1 to 4 mm [32]. This is a vertical structure with a high aspect ratio, features that are challenging to fabricate by microfabrication techniques. Another aspect to be taken into account is sweat gland density, which differs greatly across the body, but ranges between 100 and 550 glands/cm^2^ [30]. 

The typical sweat rates range from 1 to 20 nL/min·gland, according to Sonner et al. [32]. Garcia-Cordero et al. [38] differentiates sweat rates between those obtained during exercise (1.5 μL/min·cm^2^, which corresponds to 7.5 nL/min·gland, assuming 200 glands per cm^2^) or during resting conditions (20 nL/min·cm^2^ corresponding to 0.1 nL/min·gland). Henkin et al. [39] provide a value of 6.25 nL/min·gland for runners. Therefore, the majority of works typically use units of nL/min·gland to predict the volume of sample available in sweat. However, there is a remarkable variation between different body zones, as shown by Smith et al. [33] (Figure 2b). Interestingly, higher sweat rates do not correlate with sweat gland density, as their activity differs greatly. One major challenge when mimicking sweat rate is providing the low flow rates of human perspiration while ensuring uniform pore activation across all the sweating area [34].

From the dimensions and sweat generation, we proposed a microfluidic equivalent to a sweat gland using Poiseuille flow [32] (Figure 2a). The secretory coil is modeled by a network of pressure sources (sweat sources) and flow resistors because the quantity of sweat added osmotically increases over distance. The ductal region can be modeled as simple fluidic resistance as it is only a sweat conduit. The fluidic resistance of a circular channel is described by Equation (2), and it is the relation between the pressure applied and the flow rate achieved (Equation (3)). Considering the dimensions of the sweat duct (L = 4 mm and d = 10 µm) and using Equations (2) and (3), we find that a pressure of 5 kPa is enough to pump sweat through the ductal region at a flow rate of 20 nL/min·gland.

(2) $$R=\;\frac{128\mu L}{\pi d^{4}}$$

where µ is the viscosity of sweat, L is the length of the region, and d is the diameter of the circular cross-section.

P = R · Q(3)

where P is the pressure differential across the region of interest, R is the fluidic resistance, and Q is the volumetric flow rate.

Due to the hydrophobic nature of the skin surface, a Laplace pressure (Equation (4)) must be overcome as the sweat meniscus emerges onto the surface. However, this pressure barrier is no longer present when the skin is completely wet. The approximated value of the Laplace barrier generated at the sweat gland, considering a 10 µm diameter for sweat gland and a 110° contact angle, is 10 kPa, which can be overcome by the sweat osmotic pressure as we have seen before.

(4) $$P_{L}=\frac{4\gamma cos\;\theta \;}{d}$$

where P_L_ is the Laplace pressure, γ is the surface tension of sweat, θ is the contact angle of sweat on the skin surface, and d is the diameter of sweat pore. 

The skin surface is slightly hydrophobic. In terms of contact angle, it oscillates between 80 to 110° [40]. This variability can be explained by the body location and the skin’s surface state. Recent measurements have confirmed its hydrophobic value with advancing contact angles of 118º [34] and of 112.9 ± 1° for dry wrist skin [41]. Regarding skin roughness, it is known for its relation with the patterns present in the skin surface [42] (Figure 2c). First, there is a primary structure, which consists of macroscopic, wide, deep lines or furrows in the range of 20 to 100 μm. Then, a secondary structure is formed by finer, shorter, and shallower (5–40 μm) lines or furrows running over several cells. Finally, the tertiary and quaternary structures are related to cell borders and do not affect the overall skin roughness [35]. The range of the skin roughness’ values is in the order of tens of microns (Figure 2d). 

## 3. Perspiration Models

The perspiration simulation works found in the literature model the sweat gland as a fluid conduit, discarding to replicate their role on sweat generation. Sweat generation, which takes place in the secretory coil, is replaced by pumping mechanisms, such as syringe pumps and hydrostatic pressure. Although syringe pumps allow for directly setting a specific flow rate and are easier to use, hydrostatic control is sometimes preferred due to its more stable response. Therefore, the models presented are focused on the transport of sweat from a fluid source to a skin-like surface. 

Most works use laser systems, specifically, CO_2_ lasers, to machine the holes that will simulate sweat gland ductal conduits. CO_2_ lasers have been extensively used in rapid manufacturing due to their high versatility and cost-effective operation and maintenance [43]. Their infrared radiation (wavelength range of 9–12 µm) is capable of removing material by thermal ablation. The features’ characteristics (width, depth, and geometry) can be tuned by controlling laser parameters, such as the power, speed, and focal distance. These benefits have made CO_2_ lasers a well-known tool in microfluidics laboratories for rapid prototyping, without the need for molds or complex equipment [44]. In particular, they are adequately suited for fabricating passing holes in a polymeric substrate, which is the case of the perspiration models listed. Section 3.2 lists other perspiration models, which may or may not be used as a wearable interface, that use alternative fabrication methods or materials.

### 3.1. Laser-Machined Membranes

**Figure 3:**
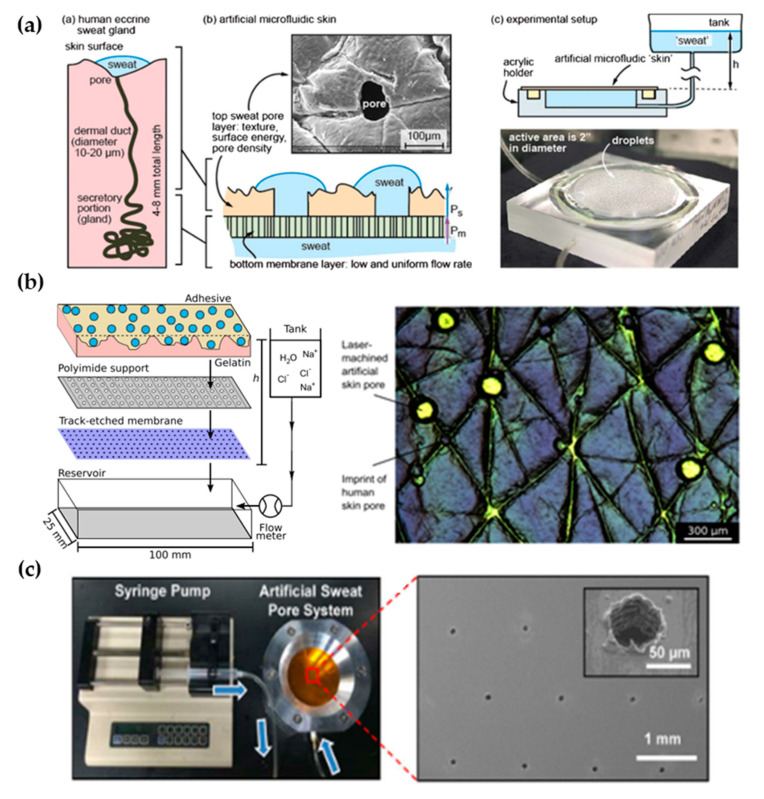
Perspiration models in literature. (**a**) Schematic of the bi-layer membrane design and its comparison with the sweat gland structure (left). Depiction of the experimental set-up and image of the integrated membrane (right). Reprinted with permission from [34]. Copyright 2013, Royal Society of Chemistry. (**b**) Scheme showing the layers of the perspiration model by Hansen et al. [45] (left). Adapted with permission from [45]. Copyright 2020, American Chemical Society. Microscopy image of the surface of the artificial skin by Eiler et al. [46] (right). Adapted with permission from [46]. Copyright 2020, Elsevier. (**c**) Experimental set-up of the artificial sweat pore system used by Koh et al. [45] and SEM image of the perforated membrane. Reprinted with permission from [45]. Copyright 2016, The American Association for the Advancement of Science.

The first work dedicated to developing a perspiration model was presented by Hou et al. [34] in 2013 (Figure 3a). The authors developed a bi-layer membrane where the bottom membrane (a commercial polycarbonate track-etched membrane) mimics the flow rate of the sweat gland while the top layer provides the proper pore density and surface texture. The bottom membrane must be hydrophilic and with pores small enough to dominate the pressure drop across the system. The top membrane is made of a combination of dry-film photoresists and a polyester substrate. Sweat glands were patterned on the top membrane by CO_2_ laser, obtaining a pore diameter of 80 microns with a pore density of 200 pores/cm^2^. Skin texture was replicated using a replica resin, achieving a faithful representation of skin texture, while wettability was compromised as the measured contact angle of the membrane differs from the one in human skin. The resulting thin membrane, with a thickness below 100 microns, was integrated into an acrylic holder, and sweat was pumped hydrostatically. The authors validated the sweat rate obtained experimentally using a flowmeter, showing a reasonable agreement with theoretical predictions. In addition, uniform activation of pores across the surface was demonstrated by visual inspection. 

The Thormann group recently adapted a similar membrane for skin adhesive testing [45,46] (Figure 3b). They obtained similar values of pore diameter and water contact angle as they used the same methodology, also reporting roughness measurements with a mechanical profilometer (obtaining an average roughness, R_a_, around 10 microns, which is on the same order of magnitude as human skin) and rheology tests. While Eiler et al. [45] used a syringe pump to set the desired sweat rate, Hansen et al. [46] used hydrostatic pumping in combination with a flowmeter. Membrane materials and fabrication steps did not differ greatly from the original work. The only variation was found in Hansen et al. [46], where the outermost layer of the membrane was replaced by a cross-linked gelatin, which hydrates during perspiration better mimics the human stratum corneum. This material modification increased the pore diameter from 87 to 250 µm due to the low compatibility of gelatin with laser processing. 

Similarly, Koh et al. [47] developed a perspiration model using pores patterned by laser on a polyimide membrane as part of their experimental set-up (Figure 3c). In this case, a single-layer membrane was used, but as their device was attached completely covering the perspiration zone, the effect of non-uniform pore activation was limited. The membrane was integrated into an aluminum chamber that served as a fluid source and it was controlled by a syringe pump, which was set at a constant flow rate of 5.5 µL/h for a harvesting surface area of 0.07 cm^2^.

Liu et al. [48] proposed a fabrication method to overcome the resolution limitation of laser-patterned membranes. The authors developed a skin phantom intended to replicate physical properties, and they included sweat ducts for a closer electrical simulation. They used gelatin to replicate the epidermis, and SU-8 photoresist for the stratum corneum. Using microfabrication techniques (lift-off), the authors fabricated a copper mask with holes mimicking sweat duct diameter on top of the structure before laser ablation. With this method, they reduced the diameter down to the 20 microns of sweat duct, but pore depth remained small in comparison to the values of sweat duct length. In fact, they studied the relation of laser parameters (duration and power of ablation) with the resulting pore depth, estimating a pore depth of hundreds of micrometers for their experimental conditions (ablation time of 0.1 s and laser power of 25 W/mm^2^). However, they achieved an average pore depth of under 60 µm. They did not integrate the skin phantom to any fluidic connection as they were interested in its mechanical and electrical properties. In Table 1, there is a summary of the characteristics of these types of perspiration models and their comparison with the reference values of human skin. 

### 3.2. Alternative Approaches 

**Figure 4:**
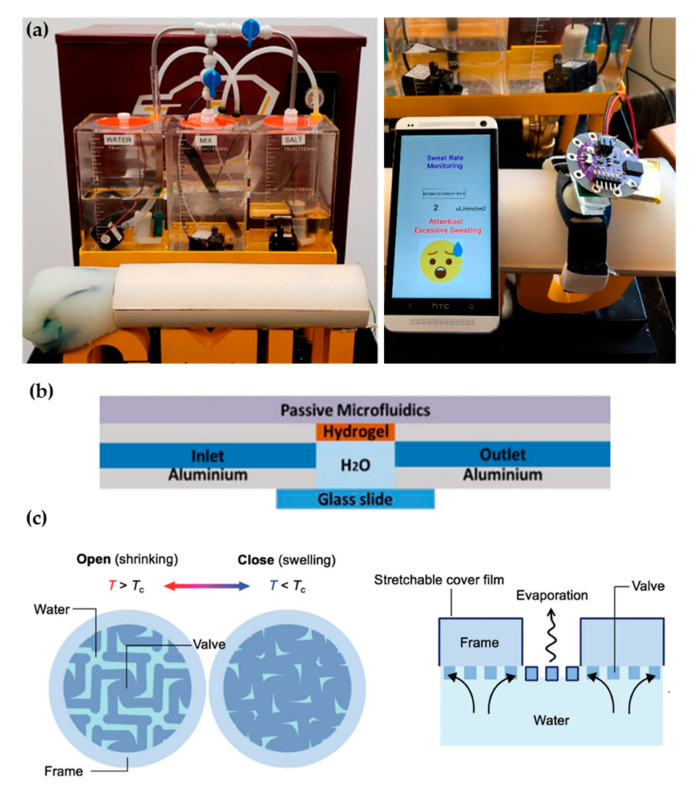
Alternative approaches for perspiration models. (**a**) Image of the sweating arm prototype developed by Brueck et al. [49] showing the arm mold and the fluid tanks for sweat solution (left). In vitro test with a wearable device that monitors sweat rate (right). Adapted with permission from [49]. Copyright 2019, MDPI. (**b**) Schematic cross-section of the artificial skin used by Garcia-Cordero et al. [52]. Adapted with permission from [52]. Copyright 2018, IEEE. (**c**) Schematic of the artificial perspiration membrane for heat dissipation developed by Kim et al. [53]. Adapted with permission from [53]. Copyright 2020, John Wiley and Sons.

Recent proposals of perspiration models are not reduced solely to laser-machined membranes. With a methodology similar to thermal manikins, Brueck et al. [49] designed an arm mold with casted silicone, which was coupled with a complex electronic system capable to control the desired sweat rate dispensed by a peristaltic pump (from 1 to 500 µL/min) and the salt concentration of the emerging sweat (from 10 to 200 mM) (Figure 4a). Details on the fabrication of the sweat pores are not given in the publication, probably opting for a system of pipes and holes similar to the one used in thermal manikins. Characterization of the flow rates achievable is validated experimentally by gravimetry.

Alternative fabrication methods have also been used for sweat gland fabrication such as the additive manufacturing by 3D printing proposed by Turcin et al. [50]. The authors fabricated a shell of a torso by fused deposition modelling (FDM) to integrate a sweating device manufactured by stereolithography (SLA). SLA, where the polymerization of a resin takes place by ultraviolet radiation, was chosen for its good surface finish of the products and resolution. Although it has been shown that SLA can arrive at the dimensions required for the sweat gland [51], the sweating device has a minimum diameter of 0.55 mm, which is much larger than the actual sweat gland diameter. This structure could be adopted as an alternative way to distribute sweat generation in a thermal manikin, but its application in sweat wearable testing would be limited. 

Hydrogels offer the possibility to recreate sweating directly through their micrometric porous structure. Garcia-Cordero et al. [52] used an agarose hydrogel as a skin-like fluidic interface to their microfluidic wearable device (Figure 4b). The hydrogel was integrated into an aluminum chamber where sweat was pumped, and it diffused through the hydrogel. Although hydrogels are a good substitute for a soft tissue such as the skin, their intricate three-dimensional pore structure does not correspond with the sweat gland structure.

Kim et al. [53] also used a hydrogel to fabricate an artificial perspiration membrane. However, their aim was to create a refrigeration system inspired in the sweating mechanism. The thermoresponsive hydrogel used served as a valve to regulate evaporation rate at the interface depending on temperature (Figure 4c). When the temperature is higher than the critical temperature (T_c_), the hydrogel shrinks, producing an enlargement in the evaporation area. This way, the evaporation and cooling effect can be regulated as a function of the temperature. This example further illustrates the wide variety of characteristics and purposes of skin models.

## 4. Perspectives for Microfluidic Wearable Technology

Sweat monitoring has recently gained relevance, shown by the multitude of new sweat wearable devices proposed each year [27,28]. Most of them use microfluidics in order to collect sweat from the skin and transport it to sensors in a controlled way [29]. The characterization of these devices consists in the conventional methodology of microfluidics laboratory (direct connection to a pumping mechanism), followed by in vivo testing with a small group of volunteers. The perspiration models presented before aim to smooth the path between them by adding an intermediate step with a realistic artificial skin setup, capable of detecting possible failures before running high effort-demanding human trials. 

**Figure 5:**
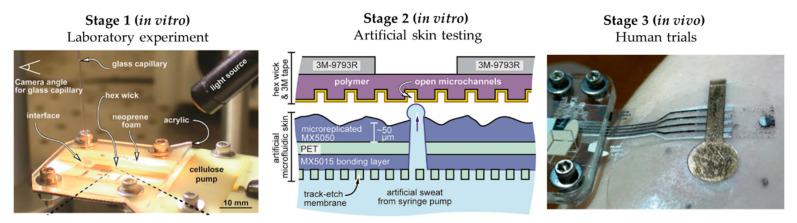
Workflow proposed for microfluidic sweat wearable characterization, using Twine et al.’s [54] work as an example. Initial laboratory experiments (Stage 1) for proof-of-concept, followed by the use of artificial skin (Stage 2) for more realistic in vitro testing for device optimization prior to human trials (Stage 3) for final validation. Adapted with permission from [54]. Copyright 2018, Royal Society of Chemistry.

The literature shows some examples of this workflow, although these examples are limited. Twine et al. [54] adapted the artificial skin developed by Hou et al. [34] to test the capillary dynamics of open nanofluidic channels (Figure 5). Initial studies were performed using a glass capillary to deliver the low volumes of fluid into the hex wick-shaped channels. The intermediate perspiration model allowed them to better mimic the collection conditions that will be faced during human testing, but under a controlled environment. Koh et al. [47] also used their perspiration model to confirm the viability of their microfluidic device for sweat sampling in aspects such as possible sweat leakage or the quantitative correlation of sweat collected. Similarly, the arm mold that Brueck et al. [49] developed was used as a with their sweat rate wearable that was previously published [55], as shown in Figure 4a, or the hydrogel interface used in Garcia-Cordero et al. [52].

Unfortunately, most works do not perform any type of testing with perspiration models. However, the complexity of the microfluidic sweat sampling methods does not cease to increase, with the use of passive and active microfluidic elements or novel materials and mechanisms. Examples include the chrono-sampling by sequential passive valves or the chemesthetic alerting system proposed by Rogers’ group [56,57]. In this scenario, there is a large step in going from the usual laboratory experiments to human trials, where the high variability of in vivo testing can distort the extracted conclusions at an initial stage. A skin-like fluidic interface (perspiration model) could facilitate the validation of these systems under controlled conditions, making the optimization process before body trials easier and faster.

## 5. Concluding Remarks

Laser-machined membranes are the predominant approach to develop skin models for perspiration simulation. CO_2_ laser is the laser system used, and it is an equipment easily available in any engineering laboratory. Researchers also use thin polymeric layers, such as polyester or polyimide, which can be machined using this approach. The resulting drilled membranes do not overpass 0.1 mm in thickness, and the most complete ones include a skin replica layer to mimic skin surface texture. Replicating sweat gland density is not a problem and it can be changed at will during laser patterning. To achieve a better flow control at low sweat rates, some works used a second commercial membrane with higher fluidic resistance than the skin-like layer. 

Although laser-machined membranes have shown a high reproducibility of human sweat rates, there are some limitations to their strategy. First, the resolution needed to replicate sweat gland ducts is difficult achieve with CO_2_ laser. A highly controlled system is required to perform such tasks, which is not generally the case in most laboratory facilities. In addition, laser-drilling limits the application on very thin membranes (cone-shaped hole), which does not correctly replicate sweat gland duct morphology in length. On the basis of the works reviewed in this article, we found that most works reached 60–80 µm in pore diameter but in membranes of less than 100 µm. The work closer to the range of diameter found in the ductal region is the 20 µm diameter pore of Liu et al. [48], but this involved a copper mask to direct laser ablation to the desired apertures. However, in their case, pore depth remained far from the values of actual sweat glands. These examples show the well-known challenges of fabricating high aspect ratio features at the micrometric scale, especially for laser systems. Future innovative solutions may overcome this constraint.

In terms of materials, the use of skin replicas for surface texture is detrimental in replicating the hydrophobic nature of skin. A balance between these two characteristics must be achieved, as wettability of skin is crucial to reflect perspiration and its interaction with any wearable fluidic system. Moreover, the use of a secondary commercial membrane as fluid resistor complicates the fabrication and integration steps. A perspiration model must be simple and easy to fabricate, with accessible technologies and techniques for any laboratory, as their purpose resides in an in vitro characterization setup for device/product testing. The final objective should be the implementation of a standardized methodology (ISO type) for the in vitro testing of microfluidic wearable sensors. 

## Figures and Tables

**Table 1:** Comparison of the key parameters of laser-machined perspiration models found in the literature with respect to human skin values.

Work	Sweat Gland Diameter (µm)	Sweat Gland Length (mm)	Sweat Gland Density (cm^−2^)	Contact Angle (°)	Roughness R_a_ (µm)	Sweat Rate (µL/min·cm^2^)	Fabrication Method	Flow Control
***Human skin* [32]**	***10–20***	***1–4***	***100–550***	***80–110* [41]**	***10–50 (RMS)* [35]**	***0.2–4***	*-*	*-*
Hou et al. [34]	80	<0.1	200	θ_a_= 76	-	0.8–5	CO_2_ laser	Hydrostatic pressure
Eiler et al. [45]	86.8 ± 17.5	<0.1	100	69.2 ± 3.6	12.1 ± 1.3	0.5–2	CO_2_ laser	Syringe pump
Hansen et al. [46]	250	<0.1	100	77.5 ± 0.8	8.4 ± 4.5	0.5–2	CO_2_ laser	Hydrostatic pressure
Koh et al. [47]	60	<0.1	100	-	-	1.3	CO_2_ laser	Syringe pump
Liu et al. [48]	20 ± 3	<0.1	620	-	-	-	Lift-off + CO_2_ laser	-
